# Application of visual placement of a nasojejunal indwelling feeding tube in intensive care unit patients receiving mechanical ventilation

**DOI:** 10.3389/fmed.2022.1022815

**Published:** 2022-11-22

**Authors:** Yuequn Chen, Xin Tian, Cheng Liu, Liqin Zhang, Yueyuan Xv, Shuang Xv

**Affiliations:** ^1^Department of Intensive Care Unit, The Fifth Affiliated Hospital of Wenzhou Medical University, Lishui Municipal Central Hospital, Lishui, China; ^2^Department of Digestive Internal Medicine, The Fifth Affiliated Hospital of Wenzhou Medical University, Lishui Municipal Central Hospital, Lishui, China; ^3^Department of Equipment Department, The Fifth Affiliated Hospital of Wenzhou Medical University, Lishui Municipal Central Hospital, Lishui, China

**Keywords:** blind placement, first placement of feeding tube, intensive care unit patients, visual placement, nasojejunal dwelling feeding tube, mechanical ventilation

## Abstract

**Background:**

Compared with nasogastric nutrition, nasojejunal nutrition may prevent some complications of critically ill patients by maintaining better nutritional status, and blind placement of nasojejunal dwelling feeding tubes is widely used. However, the visual placement seems to be safer and more effective than the blind placement, and is still seldom reported.

**Objective:**

We tried to develop visual placement of a nasojejunal feeding tube in intensive care unit patients.

**Methods:**

A total of 122 patients receiving mechanical ventilation were admitted to the Department of Critical Care Medicine of the Fifth Affiliated Hospital of Wenzhou Medical University and received the placement of nasojejunal feeding tubes. These patients were randomly and evenly assigned into two groups, one group receiving visual placement of nasojejunal dwelling feeding tubes and another group receiving blind placement. Actual tube placement was confirmed by X-ray. The primary outcome included the success rates of first placement of feeding tubes. The secondary outcome included the time of tube placement, complications, the total cost, heart rates and respiratory rates.

**Results:**

The primary outcome showed that the success rates of first placement were 96.70% (59 cases/61 cases) in the visual placement group, and two cases failed due to pyloric stenosis and gastroparesis. The success rates were 83.6% (51 cases/61 cases) in the blind placement group and 10 cases failed due to either wrong placement or retrograde tube migration. The success rates in the visual placement group were higher than that in the blind placement group (*P* = 0.015). The secondary outcome showed that the time of tube placement in the visual placement group was shorter than that in the blind placement group (*P* < 0.0001). The cost of tube placement in the visual placement group was higher than that in the blind placement group (*P* < 0.0001). The statistical differences in complications, heart and respiratory rates were insignificant between the two groups (*P* > 0.05).

**Conclusion:**

Compared with the blind placement, the visual placement shortened the time of nasojejunal tube placement and increased success rates of first placement. The visual placement was more efficient, easy to operate, safe, and has potential clinical applications.

## Introduction

Early enteral nutrition plays a crucial role in the outcome and prognosis of critically ill patients (1, 2). Critically ill patients have acute organ dysfunction, which can lead to significant morbidity or mortality. These patients are best treated in the intensive care unit (ICU) but have the difficulty to get adequate nutrition orally. Nasogastric (NG) tube is passed through the nose, down through throat, and into the stomach, and commonly used during enteral nutrition. The patients cannot be administered by a gastric tube due to the risk factors, such as gastric motility disorder, gastric dysfunction, and or high risk of aspiration (3–5). Compared with NG tube, nasojejunal (NJ) tube is put in through the nose, goes through the stomach and ends in the jejunum. NJ tube may effectively reduce such complications, and better contribute to maintain nutritional status (6). The patients who choose NJ indwelling feeding tube for enteral nutrition therapy are significantly more advantageous than those using a NG indwelling feeding tube. For those critically ill patients at high risk of aspiration, the guidelines recommend post-pyloric feeding (7). Post-pyloric feeding can better improve nutritional status, and reduce gastric retention and inhalation (8). NJ indwelling feeding tube seems to be a safer and more effective choice as compared to NG indwelling feeding one among critical patients (8, 9). Furthermore, the overall success rates of first placement for NG feeding tube sometimes were <70% (10).

Among the intubation techniques of NJ tube, blind bedside placement of feeding tubes is more used in critically ill patients (11, 12). The insertion is involved esophageal, gastric, and postpyloric placement. The key to the successful placement should be certain for the tube tip position at each stage before proceeding to the next (13). Blind placement of NJ feeding tubes is uneasy and many radiographs are required to judge placement, and the placement sometimes cannot be confirmed. The success rates of tube placement, and the time of tube placement still restrict the early implementation of enteral nutrition in critically ill patients (14). Normally, the success rates for blind placement range from 15 to 97% (15). Some studies reported that after a series of training and assessment, the success rate of blind placement can reach more than 80%, but the first success rates and placement time is still long (16). Blind intubation has the risk of misplacement into the airway. A study showed that among 748 critically ill patients, 14 were inserted into the airway by NJ feeding tube, accounting for 2% of the total number of patients (11). For the patients with pulmonary diseases, such as chronic obstructive pulmonary disease, once the pneumothorax is caused by the error of intubation, it will be a fatal operation. Repeated intubation will cause damage to nasal mucosa, esophagus, and stomach (17). Although X-ray examination is used to determine the tube position, children and pregnant women may face the risk of radiation overexposure (17). It has been reported that the use of magnetic navigation (18) and B-ultrasound assistance (19) can improve the success rates of blind intubation, but the two techniques require more manpower and material resources, and require expensive equipment and long-term experiences.

With the continuous development of clinical medicine, visualization has become the “gold standard” (20, 21). A feeding tube system is constructed to receive the signal of the image from the endoscope on another end and to exactly control the position of the indewelling tube inside human body. In-dwelling NJ feeding tube through the visualization system can avoid the risk of NJ tubes entering the airway and fatal complications in critically ill patients. Real-time imaging through visualization can be used to observe the anatomical structure. After the catheter placement is completed, there is no need to judge the position of the NJ feeding tube through auxiliary examinations, and timely feeding can be achieved. With visual placement, the tubes can reach the ideal position compared with those with the blind placement.

Considering these points, we developed visual placement of NJ indwelling feeding tube with the inside diameter in 14Fr (French gauge) or less. The endoscope tip can be moved in two directions to guide the indwelling NJ tubes into the duodenum under visualization. After being familiar with the visual placement of feed tube process, a comparative study was conducted with blind placement, and the position of the feeding tube was determined by X-ray after the tube intubation.

## Materials and methods

### Participants

From January 2020 to December 2021, a total of 122 patients receiving mechanical ventilation were admitted to the intensive care department and required NJ tubes, and selected in this study. This study was approved by the Hospital Ethics Committee of The Fifth Affiliated Hospital of Wenzhou Medical University, Lishui Central Hospital (Lishui 323000, China). Before the placement of NJ tubes, the patients were fully informed of the risk of tube intubation, and the patients signed the informed consent and participated this study voluntarily.

### Inclusion criteria

All the patients were more than 18 years, unable to eat orally, receiving mechanical ventilation, and needing enteral nutrition. There were feeding intolerance, regurgitation and aspiration. They had swallowing dysfunction, and high risk of aspiration. They would receive tracheal intubation or tracheotomy.

### Exclusion criteria

The following patients were excluded from this study: (1) they were conscious and it was not necessary to receive endotracheal intubation; (2) they had esophageal varices, intestinal obstruction, intestinal perforation, intestinal absorption disorders, upper gastrointestinal bleeding, pyloric obstruction, esophagus, stomach, duodenal lesions, and other most gastrointestinal disorders that affected the experiment and surgery; (3) they had other tube placement contraindications of NJ tubes; (4) the patient's family members (or patient legal representatives) did not agree to sign the informed consent form.

### Visual placement of NJ tubes

**Figure 1:**
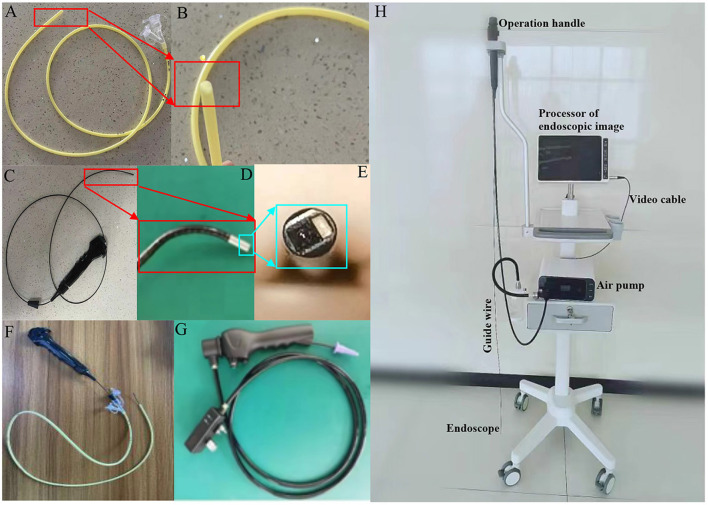
The system for endoscopic placement of nasojejunal feeding tubes. **(A)** A nasojejunal feeding tube. **(B)** The bullet shaped tip of the feeding tube. **(C)** Operable handle, guide wire, and endoscope connected with another end of the wire. **(D)** The end of guide wire and endoscope. **(E)** The tip structure of an endoscope. **(F)** The assembly is composed of an operable handle, guide wire, an endoscope, and a nasojejunal feeding tube. **(G)** Power cord and an operation handle. **(H)** The instrument of for endoscopic placement of feeding tubes.

A feeding tube (model, WII-4.7-1200 mm; Jiangsu Jianzhiyuan Medical Equipment Technology Co., Ltd., Yangzhou, China) and electronic NJ tubes endoscope from Jiangsu Jianzhiyuan Medical Equipment Technology Co., Ltd. (model, VGT-200, GT-300, Yangzhou, China) were used. The system for visual placement of NJ tubes consists of the following parts: a NJ feeding tube (Figure 1A) has the bullet shaped tip (Figure 1B). An operable handle is connected with the guide wires, and an endoscope is connected with the wires on another end (Figures 1C,D). The end of the guide wires has a tiny snake-shaped metal steer, which can be adjusted to guide the direction of endoscope (Figure 1D, the supporting video with the title: direction control). The steer also promotes the tube progression by the peristalsis. The endoscope consists of a three-layer structure (the inner and outer layers are made of polyether-block-amide, and the middle layer is woven with 304 stainless steel wire). The front end is the complementary metal-oxide semi-conductor (CMOS) image sensor and lens, and there is a tiny light-emitting diode (LED) lamp. CMOS and LED wires, and two traction steel wires are all in the sheath tube (Figure 1E). The guide wires are placed in the feeding tube and controlled by an operation handle (Figure 1F). The handle is also connected with a power cord (Figure 1G). Endoscopic image can be visualized *via* a display screen (Figure 1H). The whole process was provided *via* the supporting video (title: Visual techniques). The endoscope (its diameter is <2.6 mm) can be placed in a standard 14Fr NJ tubes, and the tube can reach the target position through the endoscope at the front end of the NJ tubes (Figure 1E). The lens of the endoscope maintains a clear view through the cold light source, and can be self-cleaned by water injection. The other end of the endoscope has a wireless real-time image, which guides the placement of NJ tubes and is obtained *via* the display screen. In this study, the operation was completed by a physician alone.

### Patients grouping

All patients were evenly assigned into two groups according to different tube placements: the patients received visual placement of NJ tubes were assigned as the VG group and the patients received blind placement of NJ tubes as the CG group. The operator was senior physician with rich experiences in ICU. The operator had many years of experiences in the operation of blind placement of NJ tubes, and was proficient in the instruction of the Corpak 10-10-10 protocol (200 cases of NJ tubes) according to a previous report (22). Both methods were performed by the same operator to avoid other confounding factors.

In the CG group, the Corflo NJ feeding tube was used (Corpak MedSystems, Inc., Wheeling, IL, USA). The operator performed the tube placement according to the Corpak 10-10-10 protocol (22). The vital signs and adverse reactions of the patients were traced during the whole process. Corflo feeding tube (Model, 20-9551; Gauge, 10FR (140 cm); IncHalyard Health Inc, Atlanta, GA, USA) was placed according to the following steps: step 1, metoclopramide hydrochloride was intramuscularly injected (specification, 10 mg; Sichuan Tianfeng Pharmaceutical Co., Ltd., Chengdu, China) before 10 min of tube placement. The tip of a feeding tube was lubricated with paraffin oil (specification, medium; batch number, 2110CC0592; Henan Yadu Industrial Co., Ltd., Zhangyuan, China). The Nose Ear and Xiphoid (NEX) method was used to evaluate the length of tube insertion; Step 2, the patient was placed in a semi-horizontal position, and then the tube was inserted into the stomach through the nose at 30°; Step 3, tube was pushed forward in 5 cm after flushing the tube with 10 ml of normal saline, and adjusted by feeling the change in resistance until the tube reached 95-cm distance. The guide wire was completely withdrawn, and the tube was fixed; Step 4, abdominal X-ray examination showed that the NJ tube was located behind the pylorus, indicating successful placement.

In the VG group, the tube was placed according to the following steps: step 1, metoclopramide hydrochloride was intramuscularly injected 10 min before tube placement. The tube head and core were lubricated with paraffin oil; Step 2, the patient was placed in a semi-horizontal position, and the tube was inserted from the nose at 30°, through the nasal cavity, pharynx, esophagus, and then into the cardia through real-time imaging of the visualization device; Step 3, with the help of the real-time imaging and adjustable function of the visualization device, the tube entered the duodenum through the gastric body, gastric antrum, and pylorus. After the tube placement, the stylet was pulled out and the tube was fixed; Step 4, abdominal X-ray examination showed that the NJ tube was located behind the pylorus, indicating successful placement.

### Primary outcomes

The primary outcome measure was successful rate of postpyloric placement of the feeding tube only for one time. The time of the first placement of feeding tube refers to the time from the NJ tube entering the nasal cavity, to the removal of the guide wire or tube placement, and NJ tubes was successfully placed and confirmed by X-ray. The success rates of first placement were calculated by successful cases/total cases × 100%. In the CG group, tube placement was evaluated by using X-ray films. The result shows that the tube tip was located behind the pylorus, and was regarded as success of the tube placement. Otherwise, the placement of the tube failed; In the VG group, the tube tip was located behind the pylorus and the position was confirmed *via* X-ray too.

### Secondary outcomes

Secondary outcomes included the time needed for the feeding tube placement, complications including bleeding, asphyxia, and misplacement into the trachea during catheter placement, and the cost of tube placement including NJ tubes consumables, X-ray examination and other items. Heart rates and respiratory rates were measured by using Philips IntelliVue M40 M8003A Patient Monitor (Hewlett-Packard, Boeblingen, Germany).

### Sample size calculation

Sample size was calculated using following calculation (23).


(1) $$\begin{array}{l} n=\frac{{\left(Z_{\alpha }+Z_{\beta }\right)}^{2}\left(p_{1}q_{1}+p_{2}q_{2}\right)}{{\left(p_{1}-p_{2}\right)}^{2}} \end{array}$$

where *Z*_α_ is factor corresponding to type 1 error and assumed as 1.96 for a 2-tail test. *Z*_β_ is factor corresponding type 2 error i.e., power is taken as 80% and the value corresponding to 0.84. We retrospectively assessed the repeated endoscopy rate after visual placement of NJ tubes, and 5% patients would undergo a repeated endoscopy for feeding tube replacement because the feeding tube was located in the stomach, due to either wrong placement or retrograde tube migration. The successful rate *p*_1_is 96% and *q*_1_ = 1−*p*_1_ is 4%. Comparatively, the successful rate *p*_2_ of blind placement of NJ tubes is 78% as previously reported (24) and *q*_2_ = 1−*p*_2_ is 22%. The sample size of each arm will be 51 and the calculated sample size is increased by 20 percent to allow for withdrawal and noncompliance. Therefore, the final number is 61 for each group.

### Statistical analysis

IBM SPSS Statistics 20 (SPSS Inc., Chicago, IL, USA) was used to analyze and analyze the data. The measurement data were expressed in the form of mean values ± standard deviation (S.D.). The difference between the two groups of measurement data was compared by *t*-test. The counting data were compared by a Chi-square test. *P* < 0.05 was used as the standard for statistical significance.

## Results

### Baseline characteristics

All patients were divided into the VG group (*n* = 61) and the CG group (*n* = 61) by a random number method. The statistical differences for gender distribution, disease types and causes, Body Mass Index (BMI, a person's weight in kilograms divided by the square of height in meters), Glasgow Coma Scale (GCS, it provides a practical method for assessment of impairment of conscious level) and Acute Physiology and Chronic Health Evaluation Score II (APACHE II, it is designed to measure the severity of disease for the patients admitted to ICU) scores, and the levels of serum albumin and albumin were insignificant between the two groups (Table 1, *P* > 0.05).

**Table 1:** Baseline characteristics of the patients receiving nasojejunal indwelling feeding tube.

	**VG (*n* = 61)**	**CG (*n* = 61)**	***t*** **or χ2 values**	***P*** **-value**
Male/Female, cases	44/17	46/15	0.169	0.681
Age, years	66.98 ± 15.73	66.18 ± 13.06	1.216	0.226
BMI, kg/m^2^	18.55 ± 3.51	18.21 ± 3.50	0.543	0.588
Diseases				
Cerebrovascular accidents, cases (%)	43(70.5)	41(67.2)	0.000	0.999
Severe pancreatitis, cases (%)	2(3.3)	1(1.6)		
Cardiopulmonary resuscitation, cases (%)	3(4.9)	2(3.3)		
Multiple organ failure, cases (%)	0(0)	3(4.9)		
Severe pneumonia, cases (%)	10(16.4)	11(18)		
Others	3(4.9)	3(4.9)		
GCS scores	5.86 ± 0.74	5.81 ± 0.72	0.380	0.705
APACHE-II scores	19.37 ± 2.02	18.75 ± 2.12	1.647	0.102
Serum albumin	2.53 ± 0.35	2.45 ± 0.95	0.460	0.647
Serum Pre-albumin (PAB)	13.27 ± 5.16	14.92 ± 5.72	1.647	0.097

All patients were evenly assigned into two groups according to different tube placements: the patients received visual placement of NJ tubes were assigned as the VG group and the patients received blind placement of NJ tubes as the CG group. BMI, body mass index; GCS, Glasgow coma scale; APACHE II, The Acute Physiology and Chronic Health Evaluation. GCS score is used to assess the extent of impaired consciousness in various acute medical and trauma. APACHE II score evaluates ICU mortality according to a number of lab values and patient signs taking both acute and chronic disease into account.

### Primary outcomes

The success rates of first placement was 96.70% (59 cases/61 cases) in the VG group, and two cases failed due to pyloric stenosis and gastroparesis. The two cases succeeded after the second time of feeding tube placement. The success rates of first placement was 83.6% (51 cases/61 cases) in the CG group and 10 cases failed because the feeding tube was located in the stomach due to either wrong placement or retrograde tube migration. Among the 10 failed cases, 6 cases succeeded after the second time of feeding tube placement, 3 cases succeeded after the second time of feeding tube placement, and 1 case was unsuccessful even after the third time of feeding tube placement. success rates of first placement in the VG group was higher than that in the CG group (*P* = 0.015, Table 2).

**Table 2:** The comparison of time, success rate and cost of feeding tube placement.

**Groups**	**Cases (*n*)**	**Placement time of feeding tube (min)**	**Success rates of first placement (%)**	**Cost (RMB)**
VG	61	11.1 ± 3.9	96.7	1263.0
CG	61	33.3 ± 8.4	83.6	1054.0 ± 39.9
*P*-value		< 0.0001	0.015	< 0.0001

All patients were evenly assigned into two groups according to different tube placements: the patients received visual placement of NJ tubes were assigned as the VG group and the patients received blind placement of NJ tubes as the CG group.

### Secondary outcomes

The time of feeding tube placement in the VG group was shorter than that in the CG group (*P* < 0.0001, Table 2). The average cost of feeding tube placement in the VG group was significantly higher than that in the CG group (*P* < 0.0001, Table 2). The complications of endoscopy of feeding tube placement were similar between the two groups (*P* > 0.05, Table 3). The statistical difference for the complications was insignificant between the CG and VG groups. The results suggest that new visual equipment will not affect the complications of endoscopy during the feeding tube placement. Similarly, the heart and respiratory rates were also similar between the two groups (*P* > 0.05, Table 4). The results suggest that new visual equipment slightly affects affect the heart and respiratory rates during the feeding tube placement.

**Table 3:** Complications during tube placement between the two groups (*n*).

**Groups**	**Cases (*n*)**	**Gastrointestinal bleeding**	**Nasal bleeding**	**Misplaced airway**
VG	61	0.00 (0)	1.00 (1.63)	0.00 (0)
CG	61	0.00 (0)	1.00 (1.63)	0.00 (0)
*P*-value			>0.05	

All patients were evenly assigned into two groups according to different tube placements: the patients received visual placement of NJ tubes were assigned as the VG group and the patients received blind placement of NJ tubes as the CG group.

**Table 4:** Complications during tube placement between the two groups (*n*).

		**VG**	**CG**	***t***	***P*** **-value**
Heart rate (times/min)	Before placement	77.48 ± 9.44	77.84 ± 8.35	0.250	0.803
	After placement	78.47 ± 9.75	77.17 ± 13.56	0.604	0.547
Respiratory rate (times/min)	Before placement	17.37 ± 2.569	17.31 ± 2.313	0.131	0.896
	After placement	18.08 ± 2.147	17.58 ± 2.063	1.279	0.203

All patients were evenly assigned into two groups according to different tube placements: the patients received visual placement of NJ tubes were assigned as the VG group and the patients received blind placement of NJ tubes as the CG group. The data were presented as mean ± standard deviations.

### The examination of gastrointestine

**Figure 2:**
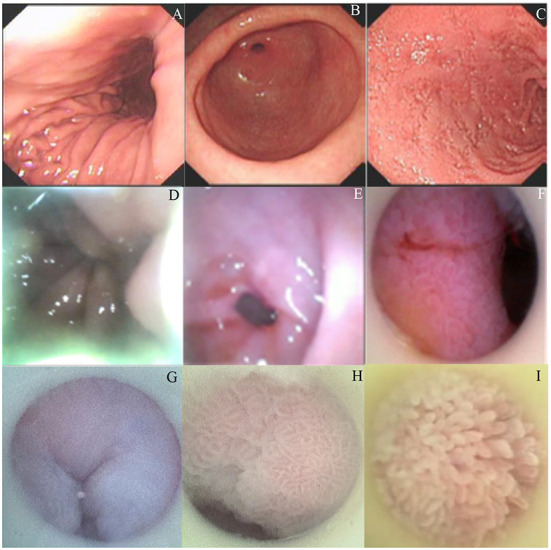
The comparison of endoscopy examination of gastrointestinal parts *via* a gastroscope and an indwelling endoscope. **(A)** Endoscopic examination of the stomach using a gastroscope. **(B)** Endoscopic examination of the pylorus using a gastroscope. **(C)** Endoscopic examination of the duodenum using a gastroscope. **(D)** Endoscopic examination of the stomach using an indwelling endoscope. **(E)** Endoscopic examination of the pylorus using an indwelling endoscope. **(F)** Endoscopic examination of the duodenum using an indwelling endoscope. **(G)** Endoscopic examination of the cardia using an indwelling endoscope. **(H)** Endoscopic examination of the gastric antrum using an indwelling endoscope. **(I)** Endoscopic examination of intestinal villi the duodenum using an indwelling endoscope.

The images of the general structure of the stomach were clearly visualized by the gastroscope (Figure 2A, stomach. Figure 2B, pylorus. Figure 2C, duodenum). The images were also obtained by using the present visual endoscope in indwelling feeding tube (Figure 2D, stomach. Figure 2E, pylorus. Figure 2F, duodenum). Most of the images obtained by this system were near to the gastrointestinal wall and the details of the gastrointestinal mucosa were very clear (Figure 2G, cardia. Figure 2H, gastric antrum. Figure 2I, intestinal villi). It would be helpful to locate and identify the direction of the pylorus by using the orientation of gastric mucosa and folds against the gastrointestinal wall. Through this study, the situation and trend of gastrointestinal mucosal changes were obtained by this system, which would be useful in the guiding and locating the position and direction of NJ tubes.

## Discussion

Most of the critically ill patients admitted to the ICU were in the state of tracheal intubation or tracheotomy, and mechanical ventilation. Such patients often took sedative and analgesic and other related drugs in the early treatment. These treatments had significant inhibitory effects on gastric peristalsis, and a large amount of nutrient solution could not be digested and absorbed immediately. Nutritional support by a gastric tube would cause insufficient nutrient intake, digestion and absorption obstacles, which increased the risk of reflux aspiration. An indwelling NJ tube was hoped to be used to improve the complications. If the patient was conscious and did not undergo tracheal intubation or tracheotomy in the ICU, they generally chose oral diet to avoid the discomfort caused by indwelling NJ tubes. Moreover, the patients who were conscious and without endotracheal intubation had poor compliance, and these patients were rarely admitted to the ICU. Consciousness can affect gastric motility and even *via* placebo (25). Compared with conscious persons, unconscious ones would likely to have delayed gastric motility. Thus, the former needed the help of NJ tubes much less than the latter. Furthermore, some conscious patients were intolerant of indwelling NJ tubes and became extremely uncooperative, or even resisted, which could significantly affect the final results. Therefore, the conscious persons were excluded from this study.

Compared with blind placement of NJ tubes, visual placement of NJ tubes significantly improved success rates of first placement, shortened the time of tube placement, and had no obvious adverse reactions and a little inconvenience. The reduction in intubation time, various auxiliary operations, radiation exposure and the damage caused to patients would be of great clinical significance for promoting nutrition and improving prognosis of critically ill patients. The placement of the 14Fr or 12Fr NJ tubes (26) with the visual device, the diameter of the endoscope should be controlled at about 2.0–2.6 mm (27), and the length of tube should be controlled at about 140–150 cm (28, 29). The bending part of endoscope camera controller guided the direction of the feeding with the help of endoscope. With the advancement of the miniaturization of visual equipment, the clinical vision can be improved. The lens has a clear field of view with the light source (the video was provided from supporting material). The situation and trend of gastrointestinal mucosal changes obtained by this system can guide and locate the position and direction of NJ tubes, and guide the intestinal tube to reach behind the pylorus. The required operating techniques and operating procedures of this system are different from those of gastroscopes.

The success rates of first placement of visual placement of NJ tubes was higher than that of blind placement of NJ tubes and time of tube placement was shorter than that of blind placement of NJ tubes. The workload of medical staff for placing the feeding tube with the visualization device was significantly reduced. Because of its visualization, no other assistance was needed. Enteral nutrition could be carried out in shorter time, effectively avoiding delayed feeding, and improving the nutritional status and prognosis of patients. Theoretically, one-time success of feeding tube placement will be safer than repeated tube placement by avoiding the damage to the patient's digestive tract mucosa caused by repeatedly inserting the feeding tubes. Notably, the cost of visual placement of NJ tubes was still higher than that of blind placement of NJ tubes. The difference was mainly caused by the high operation price in visual placement of NJ tubes, which was authorized by our local government. We hope the cost will be reduced with the promotion of visual placement of NJ tubes. Theoretically, X-ray examination may be unnecessary at the end of visual placement of NJ tubes. However, for a better comparison, X-ray examination was performed in the VG group too.

In the VG group, two cases failed because of either wrong placement or retrograde tube migration. Although the structure of the digestive tract was visible using visual placement of NJ tubes, we still found some shortcomings during the operation. For example, the endoscope was only be adjusted in two directions, up and down. In order to operate more reasonably and conveniently, the endoscope is hoped to be adjusted in all directions, up and down, side to side, and back and forward. In addition, the existing machine only has the function of pumping water and inflating air. We want to add a suction device in future design, so that patients with insufficient stomach preparation and high gastrointestinal pressure. Decompression will be reached *via* the suction device (30).

Visual placement of NJ tubes is currently not widely used and related products are still lacking. Bronchofiberoscope in the ICU is used in indwelling tube in a way similar to the clamp of the gastroscope, but it has a limited field of vision, and its length limits its application (31). We tried to use this method to place the indwelling feeding tube, but we found that the method had poor visual field and could not see the general anatomy of the stomach. It was difficult to find the pylorus and was unsuitable to be used in clinical trials. Under the gastroscope, visual placement made NJ tubes reach behind the pylorus under full visibility, with a near 100% success rate. Enteral nutrition was performed immediately after the tube placement, and a skilled gastroscope physician completed the tube placement within 10–20 min. However, for critically ill patients, due to the large diameter of the gastroscope, the balloon needs to be inflated for patients who need mechanical ventilation with endotracheal intubation (30). This operation will require positive end-expiratory pressure (PEEP) (32). It is unsuitable for the patients with pulmonary edema and patients who need high PEEP support. This method is unsuitable for severe patients with cardiac insufficiency, circulatory instability, hypoxemia, and abdominal distension, etc. In addition, if the hospital does not carry out bedside gastroscopy, the risk of transporting critically ill patients to the gastroscope room is very high. The patients with esophageal strictures are unsuitable for the tube placement with the aid of gastroscope. When the gastroscope is withdrawn, the feeding tube can easily be drawn out. Although the method of inserting feeding tube guided by gastroscope is the most indwelling method, and it has not been widely used in ICU yet (33). However, its large diameter has adverse effects on critically ill patients (34, 35).

Early enteral nutrition in critically ill patients is an important treatment measure to prevent complications such as enterogenic infection (36), and to improve patient nutrition and prognosis (37, 38). It has positive significance for improving the success rate of critically ill patients. The above research shows that visual placement of NJ tubes has significant clinical application potential. Visual placement of NJ tubes can provide a guarantee for adequate nutritional support for critically ill patients in the ICU and promote the recovery and prognosis of these patients.

There are some limitations to the present study. This study had no registration because we still lacked of a double-blind experiment in the included ICU trials, which was a major concern. Meanwhile, the shortage led to bias and decreased the study reliability without a double-blind experiment. The present single-center study did not allow for improvement of reproducibility, generalizability, as well as availability of the special equipment and clinical ICU patients. Obviously, the equipment was only tried to be applied to ICU patients with limited treatment and still needs significant improvement. Finally, its utilization in other patient population still needs mining. Gastrointestinal dysfunction was absent in all the patients receiving mechanical ventilation in this study. Therefore, this criterion limits the population who may be suitable to receive visual placement of NJ tubes. The work should be done in the future investigations.

## Future improvements

Air insufflation is the most commonly used in standard endoscopic settings. However, later swelling of the bowel often causes abdominal pain because the air is difficult to be absorbed. CO_2_ has been increasingly used for insufflation during endoscopy because of its safety and better tolerance with few side effects (39). CO_2_ insufflation causes lower abdominal discomfort because it can be quickly reabsorbed by human body (40). Thus, developing of the present equipment with CO_2_ insufflation will be of greatly helped to ICU patients.

The patients who cannot get enough nutrition through eating will rely heavily on indwelling feeding tube, which can be placed well with the help of the endoscope (41). Bacterial colonization of reusable endoscopes can leads to following infectious outbreaks and still is an important issue although many efforts have been tried to prevent infection (42). Obviously, developing a single-use device with an endoscope can certainly eliminate the potential for device-associated outbreaks.

## Conclusion

Compared with the blind placement of NJ tubes, the visual placement shows more potential clinical application by shortening the time of feeding tube placement, lowering the cost of the operation, increasing success rates of first placement. Visual placement of NJ tubes is more efficient, easier to operate, safer, and hoped to widely developed in clinical application in ICU patients. To reduce its side effects caused by air insufflation and possible bacterial infection caused by the reusable equipment, visual placement of NJ tubes still needs a significant improvement by developing CO_2_ insufflation and a single-use device.

## Data availability statement

The original contributions presented in the study are included in the article/Supplementary material, further inquiries can be directed to the corresponding author.

## Ethics statement

The studies involving human participants were reviewed and approved by the Fifth Affiliated Hospital of Wenzhou Medical University. Written informed consent to participate in this study was provided by the participants' legal guardian/next of kin. Written informed consent was obtained from the individual(s), and minor(s)' legal guardian/next of kin, for the publication of any potentially identifiable images or data included in this article.

## Author contributions

YC, XT, and CL: conceptualization, methodology, and software. LZ and YX: data collection, analysis, and writing-original draft preparation. SX: visualization, investigation, supervision, reviewing, and editing. All authors contributed to the article and approved the submitted version.

## Conflict of interest

The authors declare that the research was conducted in the absence of any commercial or financial relationships that could be construed as a potential conflict of interest.

## Publisher's note

All claims expressed in this article are solely those of the authors and do not necessarily represent those of their affiliated organizations, or those of the publisher, the editors and the reviewers. Any product that may be evaluated in this article, or claim that may be made by its manufacturer, is not guaranteed or endorsed by the publisher.

## References

[1] Reintam Blaser A Starkopf J Alhazzani W Berger MM Casaer MP Deane AM. Early enteral nutrition in critically ill patients: ESICM clinical practice guidelines. Intensive Care Med. (2017) 43:380–98. 10.1007/s00134-016-4665-0 28168570 PMC5323492

[2] Arabi YM Reintam Blaser A Preiser J-C. Less is more in nutrition: critically ill patients are starving but not hungry. Intensive Care Med. (2019) 45:1629–31. 10.1007/s00134-019-05765-0 31531714

[3] Smith AL Santa Ana CA Fordtran JS Guileyardo JM editors. Deaths associated with insertion of nasogastric tubes for enteral nutrition in the medical intensive care unit: clinical and autopsy findings. Proc. (2018) 31:310–6. 10.1080/08998280.2018.1459400 29904295 PMC5997084

[4] Turner AD Hamilton SM Callif C Ariagno KA Arena AE Mehta NM. Bedside postpyloric tube placement and enteral nutrition delivery in the pediatric intensive care unit. Nutr Clin Pract. (2020) 35:299–305. 10.1002/ncp.10452 31990093

[5] Sandhu KK. Placement of feeding tube jejunostomy. In:Scott-Conner CEH Kaiser AM Nguyen NT Sarpel U Sugg SL, editors. Chassin's Operative Strategy in General Surgery. Cham: Springer (2022). p. 367–72. 10.1007/978-3-030-81415-1_49

[6] Chen G Wang B Liang F Sun K Liu G. Pneumonia in severe acute stroke patients fed by nasojejunal versus nasogastric tube. Int J Cerebrovasc Dis Stroke. (2016) 2016:586–91. 10.3760/cma.j.issn.1673-4165.2016.07.002

[7] Leow TH Lam S Kumar B. Rare and dangerous complication of nasogastric tube insertion. BMJ Case Rep. (2020) 13:e235677. 10.1136/bcr-2020-235677 32532895 PMC7295419

[8] Liu Y Wang Y Zhang B Wang J Sun L Xiao Q. Gastric-tube versus post-pyloric feeding in critical patients: a systematic review and meta-analysis of pulmonary aspiration-and nutrition-related outcomes. Eur J Clin Nutr. (2021) 75:1337–48. 10.1038/s41430-021-00860-2 33536570

[9] Tatsumi H Akatsuka M Kazuma S Katayama Y Goto Y Monma K. Endoscopic insertion of nasojejunal feeding tube at bedside for critically ill patients: relationship between tube position and intragastric countercurrent of contrast medium. Ann Nutr Metab. (2019) 75:163–7. 10.1159/000502676 31484175

[10] Mandal M Karmakar A Basu SR. Nasogastric tube insertion in anaesthetised, intubated adult patients: a comparison between three techniques. Indian J Anaesth. (2018) 62:609. 10.4103/ija.IJA_342_18 30166656 PMC6100283

[11] Bing X Yinshan T Ying J Yingchuan S. Efficacy and safety of a modified method for blind bedside placement of post-pyloric feeding tube: a prospective preliminary clinical trial. Int J Med Res. (2021) 49:0300060521992183. 10.1177/0300060521992183 33622069 PMC7907950

[12] Wang Q Xuan Y Liu C Lu M Liu Z Chang P. Blind placement of postpyloric feeding tubes at the bedside in intensive care. Crit Care. (2021) 25:1–3. 10.1186/s13054-021-03587-5 33975642 PMC8111987

[13] Lv B Hu L Chen L Hu B Zhang Y Ye H. Blind bedside postpyloric placement of spiral tube as rescue therapy in critically ill patients: a prospective, tricentric, observational study. Crit Care. (2017) 21:1–8. 10.1186/s13054-017-1839-2 28950897 PMC5615440

[14] Zhang L Hebao S Jiao W Huang S. Improve the success rate of inserting and intubating the spiral nasojejunal tube with bare hands. Med Res. (2021) 3:6–11. 10.6913/mr.0304.02

[15] Ruetzler K Guzzella SE Tscholl DW Restin T Cribari M Turan A. Blind intubation through self-pressurized, disposable supraglottic airway laryngeal intubation masks: an international, multicenter, prospective cohort study. Anesthesiology. (2017) 127:307–16. 10.1097/ALN.0000000000001710 28570294

[16] Duggan S Egan S Smyth N Feehan S Breslin N Conlon K. Blind bedside insertion of small bowel feeding tubes. Ir J Med Sci. (2009) 178:485–9. 10.1007/s11845-009-0351-3 19430864

[17] Miyamoto K Takayasu H Katsuki S Maeda A Suzuki K Nakamura M. Blind nasogastric tube insertion performed within one minute or for a maximum of two or three attempts during resuscitation may minimize laryngopharyngeal mucosal injury: a prospective observational study. (2022). 10.21203/rs.3.rs-1601386/v1

[18] Jha P Rupp L Bonilla L Gelfond J Shah JN Meyer AD. Electromagnetic versus blind guidance of a postpyloric feeding tube in critically ill children. Pediatrics. (2020) 146:e20193773. 10.1542/peds.2019-3773 32994178

[19] Zhang Q Sun J-H Liu J-T Wang X-T Liu D-W. Placement of a jejunal feeding tube *via* an ultrasound-guided antral progressive water injection method. Chin Med J. (2018) 131:1680–5. 10.4103/0366-6999.235874 29998887 PMC6048936

[20] Aoki M Hamasaki Y Naya M Shishikura H Kato M Shibuya K. Evaluation of measures against exposure during administration of hazardous drugs through a feeding tube. Biol Pharm Bull. (2019) 42:1823–9. 10.1248/bpb.b19-00343 31685766

[21] McCutcheon KP Whittet WL Kirsten JL Fuchs JL. Feeding tube insertion and placement confirmation using electromagnetic guidance: a team review. J Parenter Enter Nutr. (2018) 42:247–54. 10.1002/jpen.1015 29505153

[22] Seres D. Nutrition support in critically ill patients: Enteral nutrition. U: UpToDate, Parsons EP ed UpToDate [Internet] Waltham, MA: UpToDate. (2019).

[23] Jagtap N Kumar JK Chavan R Basha J Tandan M Lakhtakia S. EUS versus MRCP to perform ERCP in patients with intermediate likelihood of choledocholithiasis: a randomised controlled trial. Gut. (2022) 71. 10.1055/s-0041-1724879. [Epub ahead of print]. 35144973

[24] Lai CW Barlow R Barnes M Hawthorne AB. Bedside placement of nasojejunal tubes: a randomised-controlled trial of spiral-vs straight-ended tubes. Clin Nutr. (2003) 22:267–70. 10.1016/S0261-5614(02)00210-8 12765666

[25] Meissner K. Effects of placebo interventions on gastric motility and general autonomic activity. J Psychosom Res. (2009) 66:391–8. 10.1016/j.jpsychores.2008.09.004 19379955

[26] Kim CY Dai R Wang Q Ronald J Zani S Smith TP. Jejunostomy tube insertion for enteral nutrition: comparison of outcomes after laparoscopic versus radiologic insertion. J Vasc Interv Radiol. (2020) 31:1132–8. 10.1016/j.jvir.2019.12.010 32460963 PMC7549126

[27] Yuan S-T Zhang W-H Zou L Sun J-K Liu Y Shi Q-K. Practice of novel method of bedside postpyloric tube placement in patients with coronavirus disease 2019. Critical Care. (2020) 24:1–2. 10.1186/s13054-020-02863-0 32264974 PMC7137403

[28] Chen C-x Wei Z-d Liu Y-j Cheng S-z Guan X-d. Bedside rapid placement of nasointestinal feeding tube via ultrasound-guided stylet positioning in critical COVID-19 patients. Critical Care. (2020) 24:1–2. 10.1186/s13054-020-02990-8 32552874 PMC7301623

[29] Paulsson M Jacobsson L Ahlsson F. Factors influencing breast milk fat loss during administration in the neonatal intensive care unit. Nutrients. (2021) 13:1939. 10.3390/nu13061939 34198748 PMC8228982

[30] Marks J Martin del Campo LA Gupta S Jackson T Kalil JA Wogsland A. Endoscopic management: decompression and feeding. The SAGES Manual of Foregut Surgery. (2019) 2019:837–50. 10.1007/978-3-319-96122-4_71

[31] Sohaib M Siddiqui KM Khan MF. A reliable alternative of fiberoptic bronchoscope inunanticipated difficult airway: flexible fiberoptic cystoscope. (2018) 12:158–9. 10.4103/sja.SJA_346_17 29416487 PMC5789489

[32] Eleraki S Ahmed H Abdelrahman HA Kandeel NA. The effect of different values of positive end expiratory pressure on ventilation parameters among critically ill patients. Mansoura Nursing Journal. (2021) 8:143–60. 10.21608/mnj.2021.213087

[33] Yu Y. IDDF2020-ABS-0075 optimizing the use of gastroscope for ICU patients based on machine learning model. BMJ Publishing Group. (2020) 69(Suppl 2):A36. 10.1136/gutjnl-2020-IDDF.62

[34] Blumenstein I Shastri YM Stein J. Gastroenteric tube feeding: techniques, problems and solutions. WJG. (2014) 20:8505. 10.3748/wjg.v20.i26.8505 25024606 PMC4093701

[35] Yuen JK Luk JK Chan T-C Shea Y-F Chu ST Bernacki R. Reduced pneumonia risk in advanced dementia patients on careful hand feeding compared with nasogastric tube feeding. J Am Med Dir Assoc. (2022) 23:1541–7. 10.1016/j.jamda.2022.03.011 35489380

[36] Peng Y Yuan Z Xiao G. Effects of early enteral feeding on the prevention of enterogenic infection in severely burned patients. Burns. (2001) 27:145–9. 10.1016/S0305-4179(00)00078-4 11226652

[37] Tian F Heighes PT Allingstrup MJ Doig GS. Early enteral nutrition provided within 24 hours of ICU admission: a meta-analysis of randomized controlled trials. Crit Care Med. (2018) 46:1049–56. 10.1097/CCM.0000000000003152 29629984

[38] Liu Y Zhao W Chen W Shen X Fu R Zhao Y. Effects of early enteral nutrition on immune function and prognosis of patients with sepsis on mechanical ventilation. J Intensive Care Med. (2020) 35:1053–61. 10.1177/0885066618809893 30384813

[39] Serrani M Lisotti A Spada A Sferrazza S Calvanese C Fusaroli P. CO2 vs. air insufflation in endoscopic ultrasonography: a prospective study. Endosc Int Open. (2019) 7:E317–E21. 10.1055/a-0809-4912 30834290 PMC6395089

[40] Kim SY Chung J-W Kim JH Kim YJ Kim KO Kwon KA. Carbon dioxide insufflation during endoscopic resection of large colorectal polyps can reduce post-procedure abdominal pain: a prospective, double-blind, randomized controlled trial. United European Gastroenterol J. (2018) 6:1089–98. 10.1177/2050640618776740 30228898 PMC6137594

[41] Watanabe J Kakehi E Okamoto M Ishikawa S Kataoka Y. Electromagnetic guided versus endoscopic guided postpyloric placement of nasoenteral feeding tubes. Cochrane Database Syst Rev. (2021) 10:CD013865. 10.1002/14651858.CD013865 36189639 PMC9527636

[42] Chen Y. Proposal for an Innovative Disposable Endoscope in Partnership with Summed Taiwan in 2021. Worcester: Worcester Polytechnic Institute (2021).

