# Corrigendum: Application of visual placement of a nasojejunal indwelling feeding tube in intensive care unit patients receiving mechanical ventilation

**DOI:** 10.3389/fmed.2022.1123697

**Published:** 2023-01-09

**Authors:** Yuequn Chen, Xin Tian, Cheng Liu, Liqin Zhang, Yueyuan Xv, Shuang Xv

**Affiliations:** ^1^Department of Intensive Care Unit, The Fifth Affiliated Hospital of Wenzhou Medical University, Lishui Municipal Central Hospital, Lishui, China; ^2^Department of Digestive Internal Medicine, The Fifth Affiliated Hospital of Wenzhou Medical University, Lishui Municipal Central Hospital, Lishui, China; ^3^Department of Equipment Department, The Fifth Affiliated Hospital of Wenzhou Medical University, Lishui Municipal Central Hospital, Lishui, China

**Keywords:** blind placement, first placement of feeding tube, intensive care unit patients, visual placement, nasojejunal dwelling feeding tube, mechanical ventilation

In the published article, there was an error in the legend for Figures 1A, B as published. Figure 1 describes the system used in a visible group. However, the published Figures 1A, B incorrectly shows a blind group. The corrected legend appears below.

**Figure 1:**
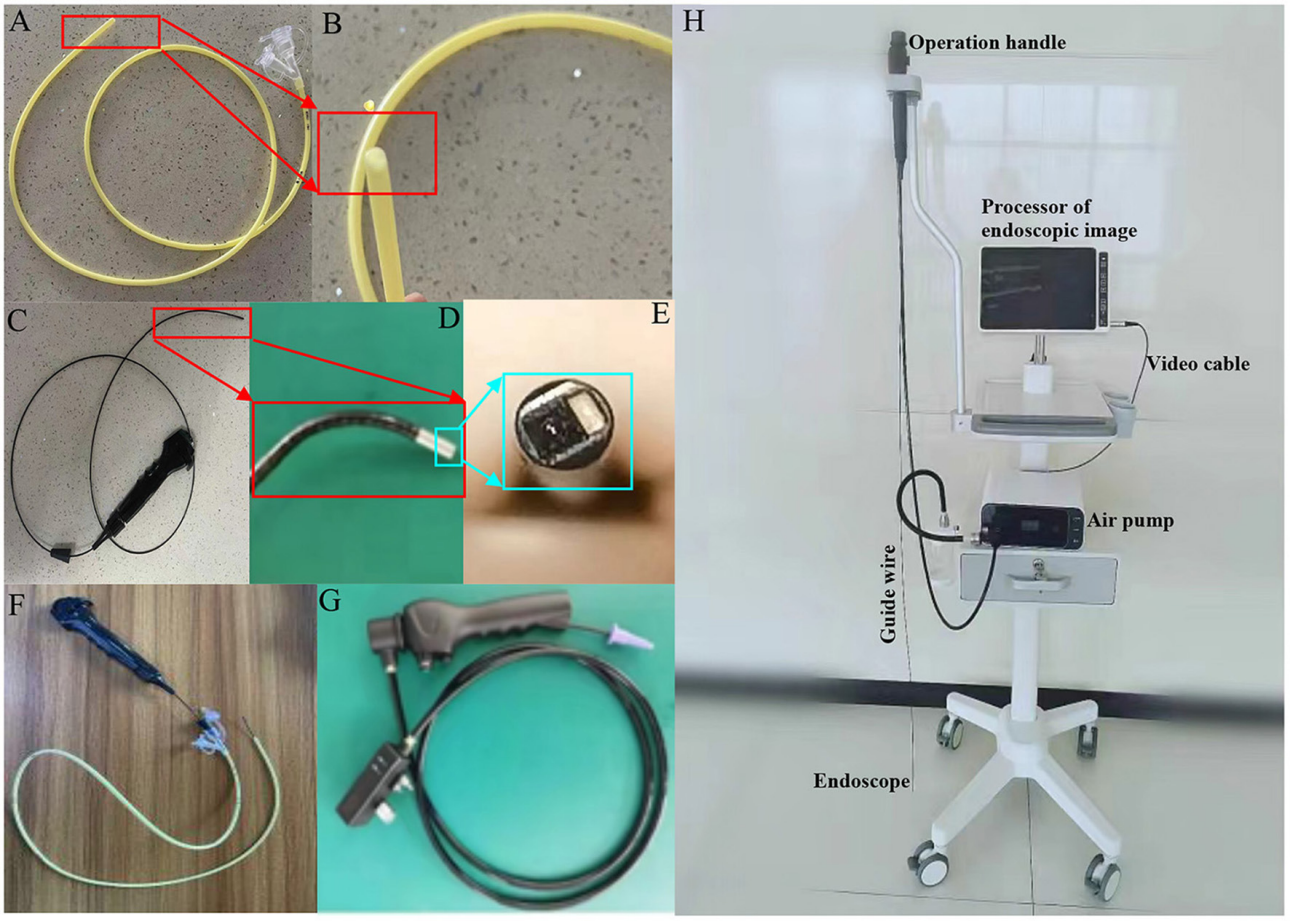
The system for endoscopic placement of nasojejunal feeding tubes. **(A)** A nasojejunal feeding tube. **(B)** The bullet shaped tip of the feeding tube. **(C)** Operable handle, guide wire, and endoscope connected with another end of the wire. **(D)** The end of guide wire and endoscope. **(E)** The tip structure of an endoscope. **(F)** The assembly is composed of an operable handle, guide wire, an endoscope, and a nasojejunal feeding tube. **(G)** Power cord and an operation handle. **(H)** The instrument of for endoscopic placement of feeding tubes.

The corrected Figure 1 appears below.

The authors apologize for this error and state that this does not change the scientific conclusions of the article in any way. The original article has been updated.

